# MIF inhibition interferes with the inflammatory and T cell-stimulatory capacity of NOD macrophages and delays autoimmune diabetes onset

**DOI:** 10.1371/journal.pone.0187455

**Published:** 2017-11-02

**Authors:** Hannelie Korf, Laura Breser, Jelter Van Hoeck, Janet Godoy, Dana P. Cook, Benoit Stijlemans, Elien De Smidt, Carolien Moyson, João Paulo Monteiro Carvalho Mori Cunha, Virginia Rivero, Conny Gysemans, Chantal Mathieu

**Affiliations:** 1 Laboratory of Clinical and Experimental Endocrinology (CEE), Department of Chronic Diseases, Metabolism and Ageing (CHROMETA), KU Leuven, Leuven, Belgium; 2 Center for Research in Clinical Biochemistry and Immunology, Department of Clinical Biochemistry, National University of Cordoba, Cordoba, Argentina; 3 Lab of Cellular and Molecular Immunology, Vrije Universiteit Brussel (VUB), Brussels, Belgium; 4 Myeloid Cell Immunology Lab, VIB Center for Inflammation Research, Brussels, Belgium; Children's Hospital Boston, UNITED STATES

## Abstract

Macrophages contribute in the initiation and progression of insulitis during type 1 diabetes (T1D). However, the mechanisms governing their recruitment into the islets as well as the manner of retention and activation are incompletely understood. Here, we investigated a role for macrophage migration inhibitory factor (MIF) and its transmembrane receptor, CD74, in the progression of T1D. Our data indicated elevated MIF concentrations especially in long-standing T1D patients and mice. Additionally, NOD mice featured increased MIF gene expression and CD74^+^ leukocyte frequencies in the pancreas. We identified F4/80^+^ macrophages as the main immune cells in the pancreas expressing CD74 and showed that MIF antagonism of NOD macrophages prevented their activation-induced cytokine production. The physiological importance was highlighted by the fact that inhibition of MIF delayed the onset of autoimmune diabetes in two different diabetogenic T cell transfer models. Mechanistically, macrophages pre-conditioned with the MIF inhibitor featured a refractory capacity to trigger T cell activation by keeping them in a naïve state. This study underlines a possible role for MIF/CD74 signaling pathways in promoting macrophage-mediated inflammation in T1D. As therapies directed at the MIF/CD74 pathway are in clinical development, new opportunities may be proposed for arresting T1D progression.

## Introduction

Type 1 diabetes (T1D) is a T cell-mediated autoimmune disease characterized by the specific destruction of insulin-producing β cells in the pancreatic islets of Langerhans. Apart from T cells, it has become increasingly clear that also other immune cells such as macrophages, dendritic cells, B cells, NK and NK-T cells as well as β cells themselves contribute towards T1D pathogenesis [1]. Macrophages in particular are recognized as the first cells to infiltrate the islets, lingering there due to abnormal adhesion molecule profiles [2–4]. They play a critical effector role in diabetes development, attributed primarily to the elevated production of inflammatory cytokines and other cytotoxic parameters [2,4–8]. This pro-inflammatory signature of T1D-associated macrophages may explain their defective capacity to clear apoptotic cells silently as well as their preferential ability to stimulate diabetogenic effector T cells rather than regulatory cells [9,10].

Factors which orchestrate the recruitment of macrophages to the islets may include a wide array of chemokines as well as macrophage migration inhibitory factor (MIF). MIF has been identified as an upstream activator of the innate immune response which mediates the recruitment and retention of monocytes/macrophages by binding to the CD74/CD44 receptor complex [11]. Its pleiotropic actions include inhibition of apoptosis [12], activation of p44/p42 MAPK signaling [13], promotion of pro-inflammatory mediators such as TNFα, NO and PGE_2_ [11,13,14], negative regulation of the immunosuppressive effects of endogenous glucocorticoids [15], and the recruitment of leukocytes to sites of inflammation [16]. In line with this, MIF has been implicated in the progression of many inflammatory and autoimmune diseases such as rheumatoid arthritis, asthma, sepsis, inflammatory bowel disease, systemic lupus erythematosus, and cancer. [17–21]. In T1D, the pathogenic contribution of MIF was reported by studies demonstrating that antibody and pharmacological inhibitor-mediated MIF neutralization had a prophylactic effect on accelerated diabetes models. Furthermore, development of multiple low-dose streptozotocin (MLD-STZ)-induced diabetes could be suppressed in mice deficient for MIF, suggesting that MIF is a key player in the development of immune-mediated diabetes [22,23]. Although these studies suggest an interesting association for MIF with T1D progression, they are limited in pinpointing the precise mechanisms for MIF in disease pathogenesis. Furthermore, it remains unclear whether the protection against disease onset by targeting MIF in these cases was not merely a result of dampening the acute inflammatory events triggered by the diabetes-inducing/accelerating chemical agents. As such the question of whether MIF targeting strategies interfere in the underlying auto-reactive T cell response is left largely unexplored. Here, we assessed the physiological relevance of MIF in spontaneous autoimmune diabetes in NOD mice and in human T1D disease by comprehensively assessing circulating and local (pancreatic) MIF levels as well as the transmembrane expression of CD74 on circulating human T1D monocytes and murine pancreatic diabetes-prone NOD macrophages. We further tested whether inhibition of MIF using a small molecule inhibitor, isoxazolines (ISO-1), could delay autoimmune diabetes onset *in vivo* and investigate possible mechanisms which may account for its disease modifying properties.

## Research design and methods

### Human subjects and samples

Control individuals were recruited from the general population at the KU LEUVEN (Leuven, Belgium). Patients with established T1D diagnosed on the basis of clinical criteria [24] were recruited from the clinical department of Endocrinology at the University Hospital Leuven. This study was approved by the institutional ethical board of the University hospital Leuven (S52697) and informed consent was obtained from every subject. Heparin-coated tubes (BD Biosciences, Erembodegem, Belgium) were used to collect peripheral blood for serum and peripheral blood mononuclear cells (PBMCs) isolation. The clinical characteristics for the patients have been summarized in S1 Table.

### Animals

C57BL/6 (C57BL/6/6NHsd; H-2b) mice were purchased from Harlan (Horst, The Netherlands) and NOD mice were bred and housed under semi-barrier conditions in our animal facility at KU LEUVEN (Leuven, Belgium). OT-II transgenic (Tg) mice which carry the MHC class II-restricted Tg TCR for OVA_323-339_ were kindly provided by Prof. Moser (ULB, Brussels, Belgium) and further bred in our animal facility. BDC2.5 TCR Tg NOD mice and NOD.SCID mice were bred from stocks originally purchased from the Jackson Laboratory (Bar Harbor, ME). Animals were maintained according to the National Institutes of Health (NIH) Guide for the Care and Use of Laboratory Animals, and all experimental procedures were approved and performed in accordance with the Ethics Committee of the KU LEUVEN (P135-2010).

### Phenotypic analysis of human T1D monocytes

PBMCs isolated from peripheral blood of human T1D patients and age-matched controls were stained with the following antibodies: CD3, CD14, CD16, CD19, CD56, CD74, and HLA-DR (all eBioscience, San Diego, CA) and matching isotype controls. Non-viable cells were excluded by using the fixable Live/Dead Yellow stain (Invitrogen) and monocytes were further defined as previously described [25]. Data acquisition was performed on a Gallios^™^ flow cytometer (Beckman Coulter, Analis, Suarlée, Belgium) and analyzed using FlowJo^™^ software (Treestar, Ashland, OR). For intracellular staining, PBMCs were stimulated with LPS and brefeldin A (eBioscience) for 18 h and then stained with the same antibody cocktail as described above followed by the addition of Cytofix/Cytoperm (eBioscience) and anti-human TNFα (eBioscience).

### Real-time quantitative polymerase chain reaction (qPCR)

Total RNA was extracted from homogenized pancreas tissue. A constant amount of RNA was reverse transcribed and the qPCR amplification reaction was performed as previously described [26]. *MIF* primers sequence: FW 5’-CGGACCGGGTCTACATCAAC-3’; RW 5’-GAACAGCGGTGCAGGTAAGTG-3’; (Eurogentec, Liège, Belgium). For mouse CD74 we used pre-designed primers from Integrated DNA Technologies (IDT), Coralville, IA using Fast SYBR^®^ Green Master Mix in combination with TaqMan^®^ Fast Universal Master Mix (Thermo Fisher Scientific/Invitrogen, Waltham, MA). All samples were normalized to the geometrical mean of ribosomal protein L27 (*rpl27*) (primers: FW 5’-GTCGAGATGGGCAAGTTCAT-3’; RW 5’-TTCTTCACGATGACGGCTTT-3’), and hydroxymethylbilane synthase (*hmbs*) (primers: FW 5’-GAAACTCTGCTTCGCTGCATT-3’; RW 5’-TGCCCATCTTTCATCACTGTATG-3’). The data were analyzed using the comparative Ct method, as described [26].

### Immunofluorescence analysis

Pancreases were cryopreserved and tissue sections were incubated with either anti-MIF (1:100; #Ab7207, Abcam, Cambridge, UK) or anti-CD74 (1:50; LN-2; #sc-6262, Santa Cruz Biotechnology, Inc., Dallas, TE) followed by Alexa Fluor^®^ 488 goat anti-rabbit or mouse IgG (1:500, Thermo Fisher Scientific/Invitrogen). Next, sections were incubated with anti-F4/80 (1:100; clone Cl:A3-1; #MCA497; Bio-rad, Hercules, CA) followed by Alexa Fluor^®^ 555 goat anti-rat IgG (1:500, Thermo Fisjer Scientific/Invitrogen). Finally, sections were stained with anti-swine insulin (1:500, #A0564; Dako, Carpinteria, CA) followed by Alexa Fluor^®^ 647 goat anti-guinea pig IgG (1:750; #106605–003; Jackson ImmunoResearch, West Grove, PA). Slides were counterstained by DAPI and mounted (Dako Faramount Aqueous Mounting Medium, Dako) and visualized using a Radiance 2001 confocal laser-scanning microscope (Bio-Rad, Hercules, CA). All acquired images were processed with Fiji/ImageJ software (NIH, Bethesda, MD).

### Diabetes intervention

New-onset diabetic splenocytes (1 × 10^7^ cells) were transferred (i.v.) into 6- to 8-week-old NOD.SCID mice. Alternatively, CD4^+^ T cells were prepared by negative selection from antigen-primed BDC2.5 TCR Tg NOD splenocytes (1 μg/mL BDC2.5 mimotope for 72 h). Activated CD4^+^ T cells (1 × 10^5^) were then transferred into NOD.SCID mice (i.v.). In both transfer models, the recipient animals were treated i.p. 5 times a week starting from the day of the transfer with 100 μg ISO-1 or vehicle control. The animals were monitored three times per week for the development of diabetes.

### Isolation and culture of murine peritoneal macrophages

Peritoneal macrophages were collected [27], and preconditioned with 20 μM MIF antagonist (ISO-1, Sigma) or vehicle control for 24h before overnight culture in the presence or absence with 10 ng/ml murine recombinant IFN-γ (Peprotech, Rocky Hill, NJ) and 1 μg/ml LPS (Sigma).

For phenotypic analysis macrophages were seeded in ultra-low attachment plates at 2 × 10^5^ cells per well before preconditioning with ISO-1 and stimulation with LPS/IFN-γ as described above. Thereafter, 2 × 10^5^ cells were labeled with the following conjugated Abs: F4/80, CD11b, I-A^d^ (clone 39-10-8 for NOD), I-A^b^ (clone AF6-120.1 for C57BL/6), CD86 and matching isotype controls (all eBioscience). Non-viable cells were excluded by using the fixable Live/Dead Yellow stain (Invitrogen). Data acquisition was performed on Gallios^™^ flow cytometer (Beckman Coulter) and the FlowJo^™^ (Treestar) software was used for data analysis.

### Circulating MIF levels and cytokine/chemokine determination

The detection of mouse and human circulating MIF concentrations was done using an anti-mouse MIF ELISA (R&D Systems, Minneapolis, MN) and a Human MIF ultra-sensitive kit (MSD, Mesoscale, Rockville, Maryland, MD) respectively. The supernatant of macrophage cultures was analyzed for cytokines/chemokines release using the Mouse Pro-inflammatory Cytokine/Chemokine V-plex kit (MSD; Mesoscale) and the human plasma samples from T1D patients and age-matched controls were analyzed using the Human Biomarker 30-plex V-plex kit (MSD; Mesoscale).

### Isolation of T cells from TCR Tg mice

Purified T cells were prepared from homogenized splenocytes from Tg mice using magnetic separation. Briefly, purified total CD4^+^ T cells (from OT-II–Tg mice or BDC2.5 TCR–Tg NOD mice) were prepared by negative selection using an Ab cocktail to CD16/CD32, CD11b, CD11c, B220, MHC class II and CD8 (all eBioscience). Contaminating, bead-bound leukocytes were removed using goat-anti-rat IgG beads (Dynabeads, Invitrogen).

### *In vitro* lymphocyte activation assays and flow cytometric analysis

CD4^+^ lymphocytes from Tg mice were co-cultured with control or ISO-1-treated macrophages with appropriate peptide (0.1–1 μg/mL) at a 1:10 macrophage / T cell ratio. Macrophage cultures were washed before addition of T cells. The co-cultures were then incubated at 37°C in 5% CO_2_ for 3 days before Ab staining and flow cytometric analysis. T cell activation was assessed by simultaneous staining for CD4, CD44 and CD69 (all eBioscience). Dead cells were excluded by using the fixable Live/Dead Yellow stain (Invitrogen). Data acquisition was performed on a Gallios^™^ flow cytometer (Beckman Coulter) and the FlowJo^™^ (Treestar) software was used for analysis.

### Statistical analysis

Survival analyses with Kaplan-Meier estimates were used to evaluate the difference in the incidence of diabetes onset, with the differences being determined by Mantel-Cox log-rank test analysis. Groups were analyzed by ANOVA (non-parametric Kruskal-Wallis test) with Dunn’s multiple comparison or with Mann-Whitney U test, as appropriate (GraphPad Prism software (Graphpad Prism, La Jolla, CA).

## Results

### Circulating MIF and its receptor during T1D

**Fig 1:**
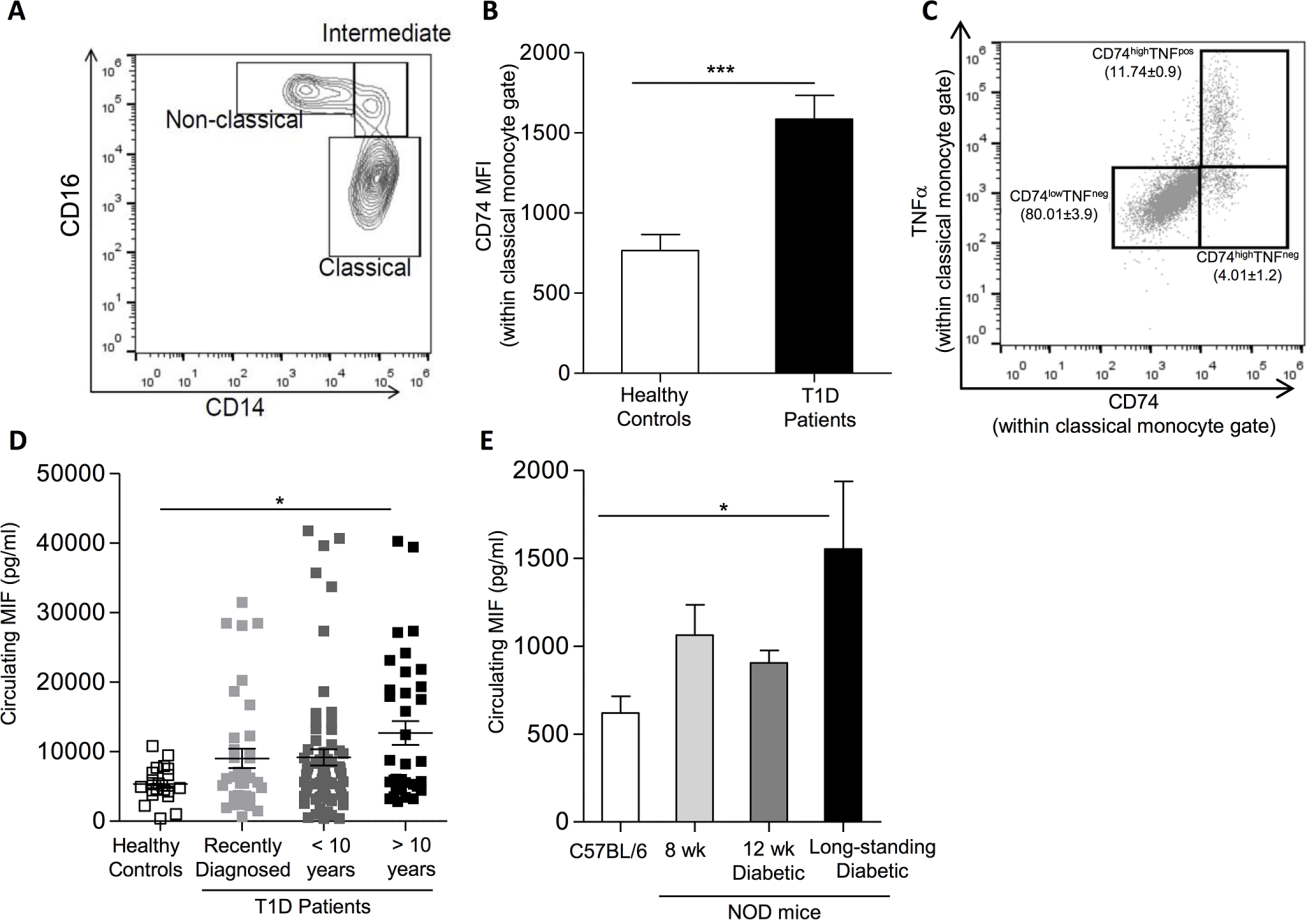
Circulating MIF and monocyte surface CD74 expression during autoimmune diabetes. (A-C) Flow cytometric analysis of human circulating monocytes from T1D patients was performed as described in the methods section. (A) Contour plot illustrating part of the gating strategy employed and how the identification of the different monocyte subsets was performed. The plot illustrates the percentage of Classical-, Intermediate- and Non-classical monocytes based on their expression of CD14 and CD16. (B) Quantification of the mean fluorescence intensity (MFI) of surface CD74 within the classical monocyte subset. The results represent mean ± SEM (n = 10) (***p≤0.005). (C) Dot plot indicating the frequency of classical monocytes producing intracellular TNFα upon activation. Notably, TNFα-positive cells also express high levels of surface CD74 (n = 10). (D) MIF levels detected in the plasma of healthy controls (open squares) (n = 20), recently diagnosed T1D patients (light grey squares) (n = 37) or of T1D patients with a disease duration of less than 10 years (dark grey squares) (n = 65) or more than 10 years (black squares) (n = 36). The symbols represent the individual samples tested (mean ± SEM) (*: p-values ≤ 0.05). (E) Circulating MIF levels as detected by ELISA in control C57BL/6 mice (white bar) (n = 6), pre-diabetic NOD mice (8 weeks) (light grey bar) (n = 10), or diabetic NOD mice (12 weeks) (dark grey bar) (n = 8) or long-standing diabetic NOD mice (16 weeks and older) (black bar) (n = 4). Results show the mean ± SEM (*: p-values ≤ 0.05).

To establish a physiological role for the MIF/CD74 pathway in T1D, we verified the expression of CD74 on circulating monocytes from healthy or (recently diagnosed and long-standing) T1D subjects. Interestingly classical monocytes of T1D patients (gating as described by Abeles *et al*. and depicted in Fig 1A) [25], presented higher surface CD74 expression compared to control subjects (Fig 1B). Investigation of the effector function of classical monocytes based on their ability to produce TNFα in response to activation, revealed that the majority of the cells displaying effector function, had more abundant surface CD74 expression (Fig 1C). In turn, we tested the circulating MIF concentrations in T1D patients and age-matched healthy controls (see S1 Table for patient characteristics). While only some recently diagnosed and long-standing T1D patients (for less than 10 years) showed elevated MIF concentrations, patients with established disease for over 10 years featured significantly higher MIF concentrations compared to healthy individuals (Fig 1D). Interestingly, circulating MIF levels positively correlated with disease duration (Spearman r = 0.253; p = 0.0092). Notably, established T1D patients did not show signs of elevated systemic inflammation as the majority of parameters within the 30-plex cytokine/chemokine biomarker panel were not significantly different than the control counterparts (S2 Table). This makes the observation of elevated MIF concentrations in some patients and particularly in the long-standing T1D patients potentially relevant. Among the few exceptions to this generalized absence of systemic inflammation was the inflammatory monocyte chemoattractant protein (MCP)-1 (S1 Fig), which was significantly higher in T1D patients than in control subjects. Importantly, both MIF and MCP-1 have previously demonstrated roles in monocyte/macrophage recruitment and retention [28,29].

We also evaluated circulating MIF concentrations at different disease stages in diabetes-prone NOD mice. Similar to the human data, significance was only reached in long-standing diabetic NOD mice compared to non-diabetes-prone control C57BL/6 animals, while the levels detected in other pre-diabetic stages (8-week-old NOD animals) as well as acute diabetics (12-week-old NOD animals) remained largely unaltered (Fig 1E).

### NOD mice featured increased MIF gene expression and CD74^+^ leukocyte frequencies in the pancreas

**Fig 2:**
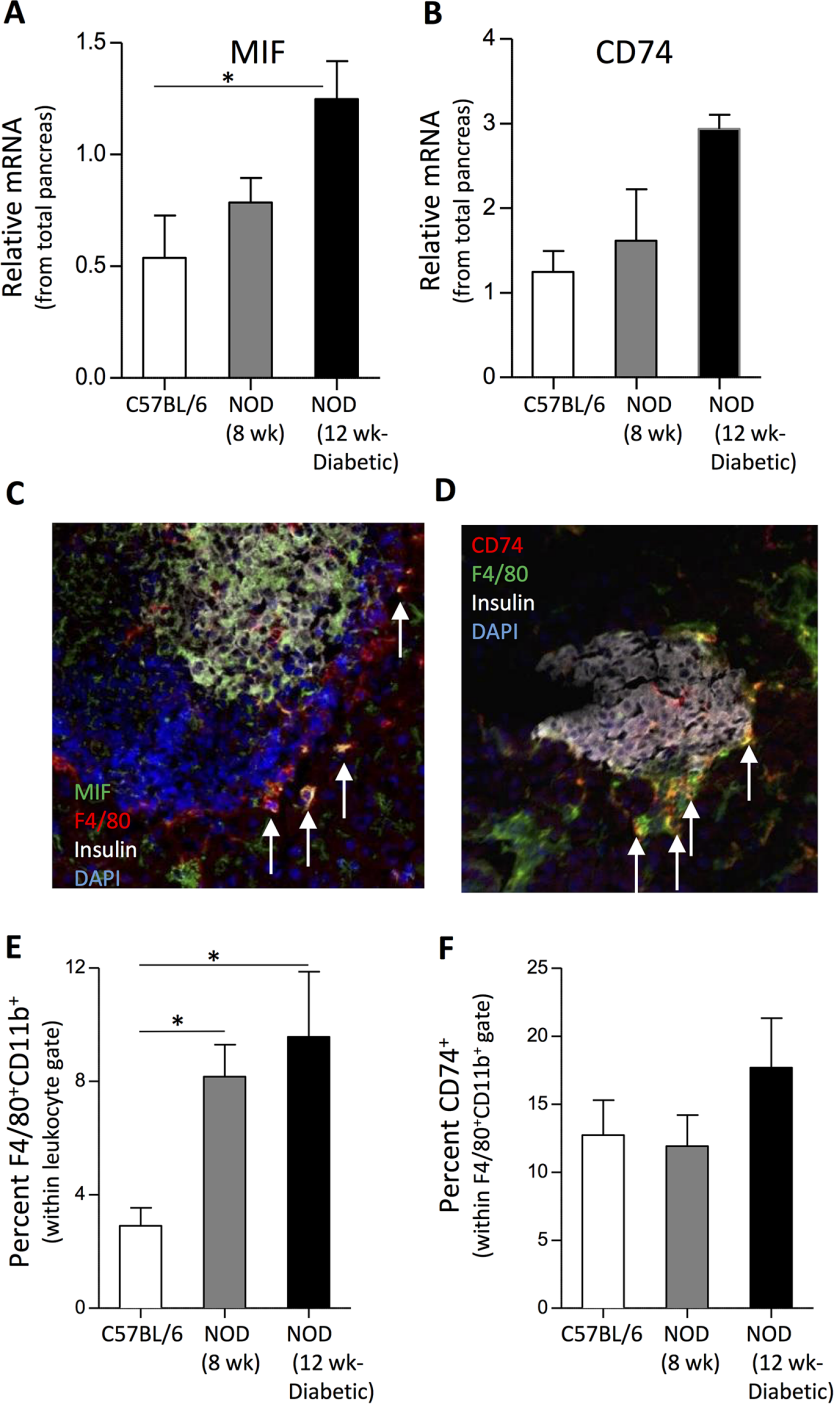
Quantification and visualization of MIF and CD74-expressing cells in the pancreas of NOD mice. Quantification of the relative MIF and CD74 mRNA levels in the total pancreas of control and NOD mice by means of real-time reverse transcription PCR (RT-PCR) (Mean ± SEM; n = 4–5) (A &B) (*: p-values ≤ 0.05). (C) Pancreatic sections of NOD mice were stained for insulin (white), MIF (green), F4/80 (red) and DNA by DAPI (blue) and analyzed with confocal microscopy at ×40 original magnification. Cells featuring a co-localization of MIF and the macrophage marker F4/80 are indicated by the white arrows. (D) Confocal microscope image of pancreatic sections of NOD mice were stained for insulin (white), CD74 (green), F4/80 (red) and DNA by DAPI (blue) (×40 original magnification). The images depicted shows one representative microphotograph documenting the presence of multiple MIF^+^- or CD74^+^ cells within the pancreatic islets of 8-week-old NOD mice. Similar results were obtained with 12-week-old acutely diabetic NOD mice (not shown). (E) Quantification of the percentage of F4/80^+^CD11b^+^ macrophages within the live leukocyte gate (mean ± SEM; n = 4–5) (*: p-values ≤ 0.05). (F) Quantification of the percentage of CD74^+^ cells within the F4/80^+^CD11b^+^ macrophage population as determined on homogenized pancreas samples by flow cytometry (mean ± SEM; n = 4–5).

Besides a systemic involvement, we investigated a role for MIF/CD74 pathways locally in the pancreas. Hereto, moderately elevated *MIF* and an upward tendency for *CD74* gene expression could be detected in homogenized pancreas material in 12-week-old animals (Fig 2A and 2B). We further verified the presence of cells expressing protein levels of MIF and its receptor, CD74, on immunostained sections of cryo-embedded pancreas tissue. Our results indicate that the source of MIF seems to be originating from multiple cell types within the islets of Langerhans including, insulin^+^ β cells and F4/80^+^ macrophages (Fig 2C) and other studies implicate also lymphocytes as possible MIF producers [30]. Cells expressing the MIF receptor, CD74, were present on F4/80^+^ cells within immune cell infiltrate of the islets of NOD mice (Fig 2D). CD74^+^ cells were absent in the pancreas sections of control animals coinciding with the absence of inflammation (data not shown). Flow cytometric analysis of homogenized pancreas revealed that the frequency of F4/80^+^CD11b^+^ macrophages in general steadily increased as NOD mice progress towards disease onset (Fig 2E). Moreover, an upward trend in the frequency of CD74^+^F4/80^+^CD11b^+^ cells in NOD diabetics compared to control mice was observed (Fig 2F). Besides macrophages and in line with previous work [31], a low abundant population of CD103^+^CD11c^+^ dendritic cells (S2A Fig) as well as a small percentage of lymphocytes expressed CD74 (S2B Fig).

### MIF antagonism dampened macrophages’ inflammatory response and expression of molecules involved in antigen presentation

**Fig 3:**
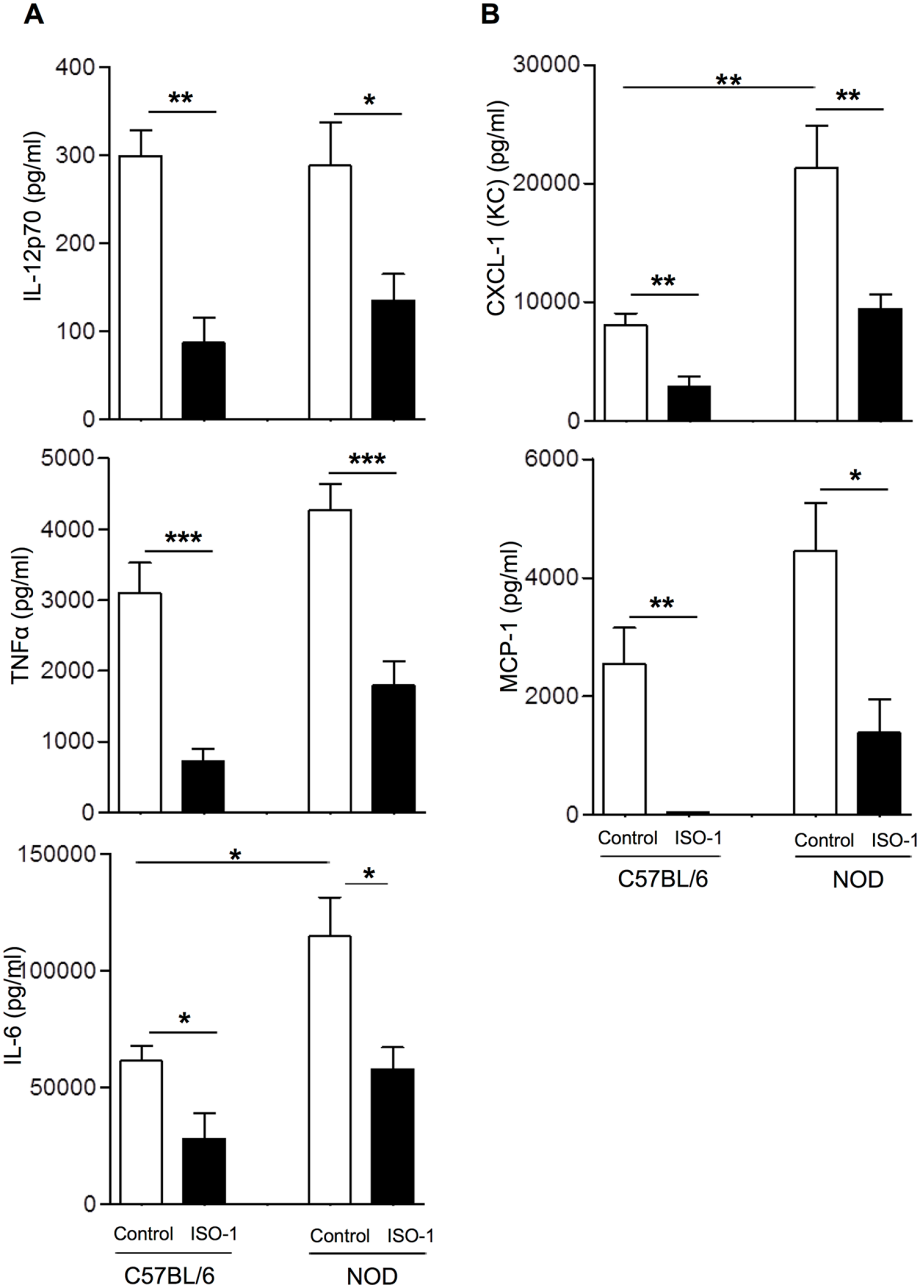
Macrophage preconditioning with ISO-1 curtails their activation-induced cytokine and chemokine production. Peritoneal macrophages isolated from C57BL/6 or NOD mice were purified by adherence as described and preconditioned with or without ISO-1 before exposure to the activation triggers. Supernatants were harvested 24 hours after addition of the activation trigger before assessment of cytokines (A) and chemokines (B) as indicated in the materials and methods section. In an unstimulated condition the levels of the depicted inflammatory parameters were low to negative (not shown). Data are presented as the mean ± SEM, (n = 8) (*, p < 0.05; **, p < 0.01, ***p<0.005).

Since NOD macrophages were implicated as one of the possible cell types to respond to the actions of MIF, we tested whether MIF antagonism could curtail their inflammatory capacity as well as expression of molecules involved in antigen presentation. As MIF targeting strategy we used the small molecule ISO-1, which inhibits MIF tautomerase activity by competing with the substrate for the catalytic site [32]. For this purpose, we pretreated peritoneal mouse macrophages with ISO-1 (20 μM) for 24 hours before exposure to LPS and IFN-γ. In line with previous findings [8], NOD macrophages produced higher protein levels of IL-6 and CXCL-1 (KC) and an upward tendency for TNFα and MCP-1, compared to non-diabetes-prone control C57BL/6 counterparts (Fig 3A and 3B). Even in this hyper-inflammatory context, pretreatment of macrophages with ISO-1 successfully counteracted or showed a clear tendency to dampen the production of the studied inflammatory parameters provoked by activation stimuli (Fig 3). In fact, ISO-1 conditioning completely abrogated the production of MCP-1 in control macrophages, an attribute which could not be completely reproduced in ISO-1-pretreated NOD macrophages. Importantly, these ISO-1-mediated effects on macrophage function could not be attributed to toxicity since we did not observe elevated cell death when macrophages had been cultured for 24 hours in the presence of the MIF antagonist (S3A Fig).

**Fig 4:**
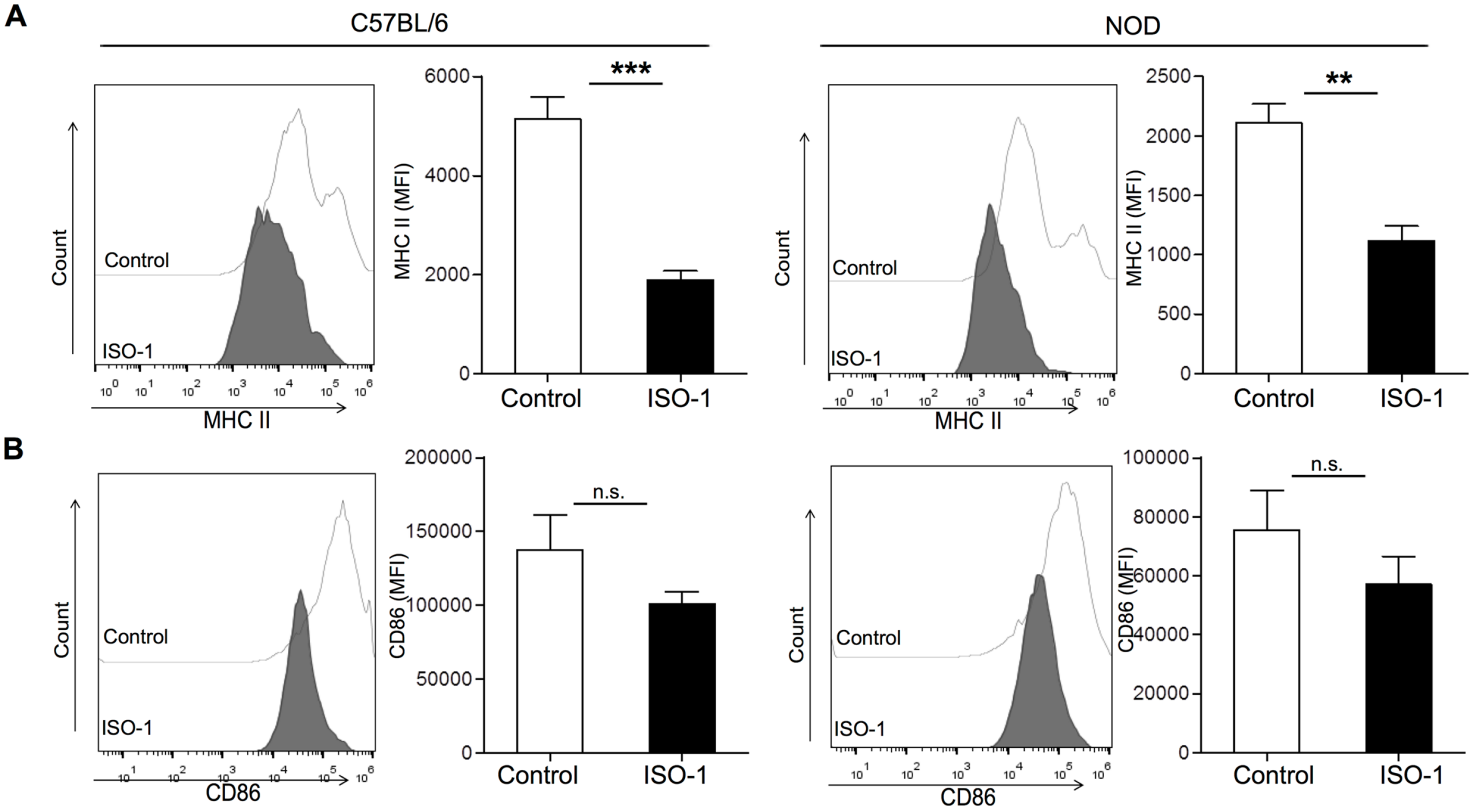
ISO-1 counteracts the activation-induced expression of MHC class II on control and NOD macrophages. (A) Surface molecule expression as analyzed by flow cytometry. Macrophages from either control or NOD mice were cultured in the presence of ISO-1 (20 μM) (dark/black) or vehicle control (light/white), followed by overnight stimulation with or without LPS/IFN-γ before staining for surface molecules involved in antigen presentation such as MHC class II (A; top row) and the co-stimulatory molecule CD86 (B; bottom row). Analysis was performed on singlet-, viable-, F4/80^+^CD11b^+^ cells. The mean fluorescence intensity (MFI) is depicted as histogram overlays (left) or bar graphs (right) (n = 4–5). The y-axis indicates the number of cells analyzed. Values represent the means ± SEM (*, p < 0.05; **, p < 0.01; ***p≤0.005).

Taken into account that ISO-1 impeded the macrophage response to inflammatory stimuli, we tested whether this modulated function would be reflected by the expression levels of molecules involved in antigen presentation. Exposure of either NOD or C57BL/6 peritoneal macrophages to ISO-1 prevented the activation-induced upregulation of MHC class II (Fig 4A). However, for the co-stimulatory molecules CD86 and CD80 this refractory action of ISO-1 was less prominent showing only a marginal inhibition for CD86 (Fig 4B), while the levels of CD80 were unaltered (data not shown).

### Inhibition of MIF delayed the development of diabetes

**Fig 5:**
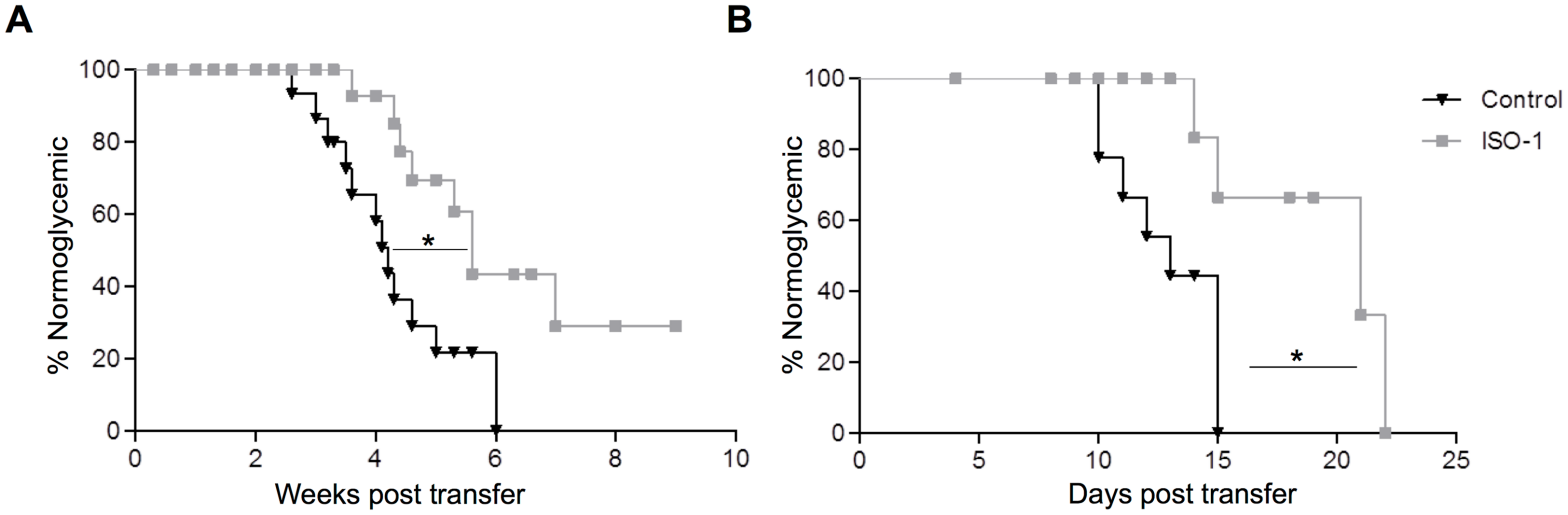
Effect of the MIF inhibitor, ISO-1, on the development of diabetes *in vivo*. (A) Percentage of normoglycemic mice following the adoptive transfer (i.v.) of splenocytes (1 × 10^7^) isolated from newly-diagnosed diabetic NOD mice into NOD.SCID mice (6–8 week-old). The recipient animals additionally received ISO-1 (100 μg; i.p.) (grey squares) (n = 9) or vehicle control (black triangles) (n = 12) five times a week and were monitored twice weekly for the development of diabetes for a total of 10 weeks. (B) Percentage of normoglycemic mice following the transfer of activated CD4^+^ T cells from BDC2.5 Tg (1 × 10^5^) mice into NOD.SCID mice. Similarly, the recipient mice received ISO-1 (100 μg; i.p.) (grey squares) (n = 4) or vehicle control (black triangles) (n = 7) five times a week and were monitored daily for the development of diabetes. In all panels, statistical significance between groups was determined by Mantel-Cox log-rank test; *p < 0.05.

In order to evaluate the potential *in vivo* relevance of MIF/CD74 pathway during T1D, we investigated whether targeting MIF could prevent diabetes transfer by autoimmune NOD splenocytes into NOD.SCID mice. In this experimental model, macrophages are promptly recruited to the pancreas following T cell transfer where they play a crucial role in amplifying T cell activation and acting as effector cells in the attack against the β cells [6,33]. Treatment of recipient mice with ISO-1 (100 μg/day; 5 times/week; i.p.) significantly delayed autoimmune diabetes onset (median onset, 4.2±0.3 vs. 5.6±0.4 weeks; Fig 5A). Importantly, also in a very aggressive variant of this diabetes model, where activated BDC2.5 TCR–Tg CD4^+^ T cells (1 × 10^5^) were transferred into NOD.SCID recipients, ISO-1 was successful in significantly delaying disease onset (median onset, 13±0.8 vs. 21±2.0 days; Fig 5B). ISO-1 treatment of recipient NOD.SCID animals significantly reduced the frequency of CD74^+^F4/80^+^CD11b^+^ macrophages, as well as in the number of lymphocytes recovered from the pancreas on day 10 post treatment initiation (S4 Fig). Of note, *in vivo* toxicity of the antagonist was excluded, since the no significant increase in cell death could be observed in animals treated daily with the MIF antagonist compared to vehicle-treated controls (S3B Fig).

### Inhibition of MIF hampered macrophages’ ability to stimulate T cells

**Fig 6:**
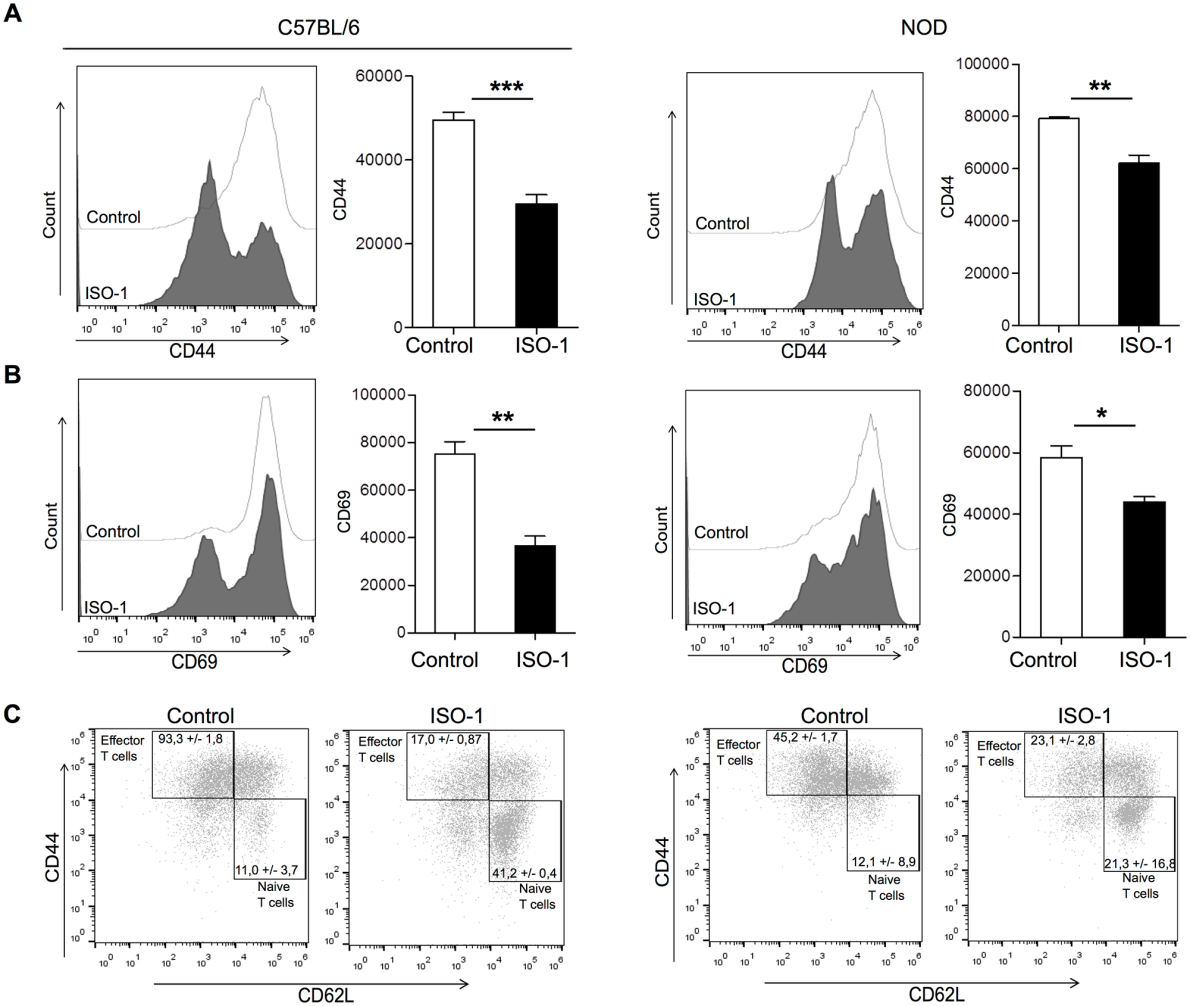
ISO-1-treated NOD- or C57BL/6-macrophages modulate T cell activation *in vitro*. (A-C) Ctr- or ISO-1-treated macrophages isolated from either C57BL/6 or NOD mice (5 × 10^4^ cells/well) were washed before addition of OVA_323-339_ peptide or BDC2.5 mimotope (1 μg/mL) and culturing together with negatively isolated CD4^+^ T cells from OT-II or BDC2.5 Tg mice (1 × 10^5^ cells/well). The activation status of the co-cultured T cells was measured by flow cytometric analysis of the mean fluorescence intensity (MFI) of CD44 and CD69 expression on viable CD4^+^ T cells after 3 days in co-culture (mean ± SEM) (n = 4) (*, p < 0.05; **, p < 0.01, ***p≤0.005). (C) To identify naïve T cells (defined as CD44^low^CD62L^high^), memory T cells (defined as CD44^high^CD62L^high^) and effector T cells (CD44^high^CD62L^low^), multi-color flow cytometry was performed and the frequencies of these populations were quantified. (C) In CD4^+^ cells, the percentages of effector T cells significantly decreased in the presence of ISO-1-conditioned macrophages, while naïve T cells remained prominently visible compared with the control condition. A p-value < 0.05 indicate significant differences between groups (n = 4). The data shown are representative of four independent experiments.

To get more insights into the mechanisms by which ISO-1 interferes with disease pathogenesis, we assessed the functional impact of ISO-1 treatment of macrophages on T cell activation in an *in vitro* system. Hereto, we co-cultured ISO-1-pretreated NOD or C57BL/6 macrophages in the presence of the corresponding antigen together with CD4^+^ T cells originated from two TCR–Tg models: BDC2.5-TCR–Tg NOD mice (expressing a Tg–TCR recognizing I-A^g7^–bound BDC2.5 mimotope in the NOD background) and OT-II–Tg mice (expressing a Tg–TCR recognizing I-A^b^–bound OVA_323–339_ peptide in the C57BL/6 background) for 72 hours. Notably in this setup there was no direct contact of the ISO-1 with the T cells as the pre-treated macrophages were extensively washed before addition of the peptide prior to addition of T cells. Corroborating the *in vivo* data, ISO-1 pre-treated macrophages clearly present a T cell hypostimulatory capacity compared to untreated control macrophages (Fig 6). This was evidenced by the decrease in the level of expression of CD44 and CD69 as indicators of T cell activation (Fig 6A and 6B). Importantly, these inhibitory actions on T cell activation were observed for both C57BL/6 and NOD macrophages.

Additional CD4^+^ T cell subset analysis revealed that naïve T cells defined as CD44^low^CD62L^high^ were markedly increased, whereas the percentages of CD44^high^CD62L^low^ effector T cells and CD44^high^CD62L^high^ memory T cells were significantly decreased when T cells had been co-cultured with ISO-1 pretreated macrophages (Fig 6C). These findings suggest that the ISO-1-pretreated macrophages blunt the activation of antigen-specific T cells by keeping them in a naïve state. Finally, T cells co-cultured with ISO-1-pretreated macrophages also exhibited an impeded capacity to trigger the effector cytokine, IFN-γ, within the T cell co-cultures (S5 Fig).

## Discussion

MIF is a pleiotropic molecule and a key mediator of many immune processes [11,34,35], and has been implicated to exacerbate inflammation in several immune disorders [36–40]. In the context of autoimmune diabetes, older reports have proposed a role for MIF as a contributing factor in disease pathogenesis in T1D [22, 23, 35, 41]. However, in most cases such a link has been established by demonstrating resistance of MIF-deficient animals to MLD-STZ challenge [22,23], whereby a non-specific chemical attack on the β cell is launched. Despite ultimately inducing hyperglycemia and insulinopenia, this model does not bear strong autoimmune features, and as such, it remains highly debatable whether MIF truly contributes to the underlying mechanisms of the autoimmune attack on the β cell. Here we provide a clear indication that MIF inhibition can counteract autoimmune diabetes in two different disease transfer models and provide unique evidence for potential mechanisms which may account for these disease modifying properties.

Along with other novel approaches [41–43], the pharmacologic targeting of MIF either by monoclonal antibodies or by small molecule antagonists for the treatment of autoimmune diseases has attracted considerable interest and new generation inhibitors are actively investigated [44,45]. We opted for a well-characterized small molecule MIF antagonist, ISO-1, for a couple of reasons. ISO-1 binds to the intrinsic tautomerase activity residing in a domain that interacts with the MIF receptor, CD74 [32,46–48], thus inhibiting both the actions of MIF as well as the signaling events triggered through CD74. Another potential advantage is its small molecular size which allows penetration into the cell neutralizing also intracellular MIF effects without inducing toxicity. However, since ISO-1 only weakly inhibits MIF binding to its receptor and possibly also because of a higher clearance rate, extremely high doses are needed for *in vivo* efficacy. Indeed, Grant et al. failed to delay diabetes development in spontaneous T1D models with a fluorinated analog of ISO-1, due to dosing or short half-life of the molecule [47]. Other studies showing successful therapeutic efficacy implemented ISO-1 at unrealistically high doses of up to 1 mg/mouse/day, which is 10 times higher than the ones used in the current study [22]. Despite the relatively low dose of ISO-1, the antagonist was successful in delaying diabetes onset in NOD.SCID recipients after adoptive transfer of diabetogenic T cells–models which are very aggressive and difficult to modulate.

We assessed the functional consequence of MIF inhibition on the *ex vivo* cytokine response of NOD macrophages as they have been implicated as the main MIF-responsive cell type. Indeed, inhibition of MIF binding to CD74 using ISO-1 suppressed the activation-induced production of pro-inflammatory cytokines (IL-12p40, TNFα, and IL-6) in NOD and control macrophages. These results are in keeping with the role for MIF as an upstream inflammatory cytokine, igniting the production of other pro-inflammatory cytokines in macrophages [11]. Besides the promotion cytokine secretion, MIF in combination with CD74 can also promote leukocyte migration to sites of inflammation via the facilitation of MAPK and PI3K/Akt signaling pathways [49,50]. In our study MIF antagonism counteracted the production of CXCL-1 (KC) and MCP-1 as respective neutrophil- and monocyte/macrophage attracting chemokines, following exposure of the macrophages to a danger signal. Of particular interest was the near complete abrogation of MCP-1 expression by the MIF inhibitor. This result is in line with previous studies implicating MCP-1 as a direct downstream target of MIF/CD74 pathways. In fact, MIF has been shown to exert its monocyte/macrophage recruitment activities through MCP-1 induction [29]. Additionally, MCP-1 over-expression in insulin-producing pancreatic β cells was shown to triggered severe insulitis and diabetes [51]. Upon binding of MIF, CD74 can form functional signal transduction complexes with CD44, CXCR4, and CXCR2 and as such targeting MIF/CD74 pathways may regulate also numerous other biological activities [50,52,53]. Although the latter aspects have not been addressed here, it would be highly interesting to determine whether targeting the CXCR4-axis, either directly or indirectly through CD74, will lead to improved therapeutic efficacy in T1D models. Notably, such strategies have been shown to be highly effective by the group of Fiorina *et al*. [54,55].

Unexpectedly, ISO-1-preconditioned C57BL/6 or NOD macrophages showed a potent capacity to interfere with the activation of OVA-reactive OT-II- or autoreactive BDC2.5 CD4^+^ T cells *in vitro*. In both cases pretreatment of macrophages with ISO-1 seemed to prevent priming of the T cells as the majority of the T cells remain in a naïve status featuring a CD62L^high^CD44^low^ phenotype, while only a small percentage convert into an effector CD62L^low^CD44^high^ phenotype. This reduced capacity of ISO-1-pretreated macrophages to trigger the transition of naïve T cells to effector T cells was further supported by the impaired production of IFN-γ, a typical effector cytokine produced by activated Thelper-1 cells. The ability of ISO-1 to impede T cell priming rather than T cell effector function may partially explain the moderate disease modifying effect of ISO-1 in the NOD.SCID transfer model, as the T cells within the donor diabetogenic cells may already exhibit an effector phenotype. Nevertheless, ISO-1 treatment succeeded in triggering a significant delay in the disease onset suggesting at least some inhibitory action also on already activated T cells or their recruitment *in vivo*. Although we observed a reduced number pancreatic CD74^+^ macrophages and lymphocytes in NOD.SCID recipient animals following 10 days of ISO-1 treatment, considering the multifunctional nature of MIF, we cannot exclude that also other mechanisms could be responsible for the disease modifying properties of MIF observed here.

Tolerance induction by macrophages could include a number of mechanisms, such as the induction of T cell hyporesponsiveness due to reduced expression of antigen presenting or costimulatory molecules, T cell silencing or deletion, but also the induction/expansion of regulatory T cells (Tregs) or Treg-promoting secreted factors [56–59]. Notably we did not observe the appearance of CD4^+^FoxP3^+^ Tregs or elevated levels of regulatory mediators such as IL-10, at the end of the three-day co-culture period (data not shown). Within the limits of our assays, our data revealed that ISO-1 pretreatment prevented the induction of MHC class II as crucial parameter involved in the antigen-presentation, while no significant inhibition of co-stimulatory molecules CD86 and CD80 could be observed. In this regard, it is important to note that CD74 has been shown to act as an MHC class II chaperone, which promotes endoplasmic reticulum exit of MHC class II molecules, directing them to endocytic compartments, preventing peptide binding in the ER, and contributing to peptide editing in the MHC class II compartment. Additionally an accessory role for CD74 was reported during T cell responses through its interactions with CD44 [60]. It is plausible that inhibition of MIF binding to its receptor also indirectly affected the normal function of CD74 molecules as participants during antigen presentation, explaining the impediment of ISO-1 preconditioned macrophages to trigger T cell activation. Taking this in consideration, an elegant study by Benedek *et al*., implemented partial MHC complexes loaded with a disease-relevant antigen to inhibit MIF/CD74 downstream signaling, an approach which successfully curtailed disease processes of autoimmune encephalomyelitis [61]. Although the approach is promising, additional work is needed to understand how blocking MIF/CD74 pathways resulted in such profound T cell inhibitory action.

In summary we provide evidence of elevated MIF levels and the frequency of CD74^+^ cells within the islets of diabetic animals and within the circulation of long-standing T1D patients. Blocking MIF/CD74 pathway not only interfered in disease onset but also hampered macrophage cytokine and chemokine responses, supporting a role for MIF as upstream regulator of innate responses. Strikingly our data also point to a role for MIF in the functional capacity of macrophages to trigger T cell activation. Such a dual action may proof very effective to intervene in chronic inflammatory diseases and the potential use for MIF neutralizing or antagonizing strategies specifically in T1D and potentially also other inflammatory disorders, warrants further investigation.

## Supporting information

S1 Fig Circulating MCP-1 determination from T1D patients. MCP-1 levels were detected in the plasma of established T1D patients (n = 46), recently diagnosed T1D patients (n = 8) and healthy controls (n = 21) as described in the method section. The symbols represent the individual samples tested and show also the mean ± SEM.
(PDF)

S2 Fig Frequencies of CD74^+^ leukocytes in the pancreases of NOD mice. Flow cytometric analysis of homogenized pancreas of NOD mice from different ages whereby the frequency of CD74^+^ cells within the CD103^+^CD11c^+^ dendritic cell-gate (A) or lymphocyte-gate (B) have been analyzed (mean ± SEM; n = 4–6).
(PDF)

S3 Fig Evaluation of *in vitro* and *in vivo* macrophage viability following treatment with ISO-1. A) Flow cytometric analysis or Live/DEAD staining on macrophages cultured for 24 hours in the presence of ISO-1. Values represent the means ± SEM (n = 4) of the percent non-viable cells. B) Flow cytometric analysis of Live/DEAD staining on macrophages within homogenized pancreas of NOD.SCID animals. The NOD.SCID recipients were adoptively transferred with activated CD4^+^ T cells obtained from BDC2.5 Tg (1 × 10^5^) and received in vivo treatment with ISO-1 as described in materials and methods. All animals were sacrificed on day 10 post treatment initiation (mean ± SEM; n = 5).
(PDF)

S4 Fig Effect of in vivo ISO-1 treatment on pancreatic immune cell infiltrate. NOD.SCID recipient animals were adoptively transferred with activated CD4^+^ T cells obtained from BDC2.5 Tg (1 × 10^5^) mice as described in the methods section. The recipient mice received ISO-1 (100 μg; i.p.) (black bars) or vehicle control (white bars) five times a week. On day 10 post treatment initiation, the percentage of CD74^+^ cells within the F4/80^+^CD11b^+^ macrophage population (A) or lymphocytes (B) were quantified in homogenized pancreas samples by flow cytometry (mean ± SEM; n = 5).
(PDF)

S5 Fig IFN-γ production by the ISO-1-pretreated macrophages- and activated T cell co-cultures. Ctr- or ISO-1-treated macrophages isolated from either C57BL/6 or NOD mice (5 × 10^4^ cells/well) were washed before addition of OVA_323-339_ peptide or BDC2.5 mimotope (1 μg/mL) and culturing together with negatively isolated CD4^+^ T cells from OT-II or BDC2.5 Tg mice (1 × 10^5^ cells/well). After 72 hours the supernatants were collected and tested with an MSD IFN-γ V-plex assay.
(PDF)

S1 Table Patient characteristics. The general and disease-associated characteristics of the T1D patients and age-matched controls used to assess circulating MIF levels.
(DOCX)

S2 Table Circulating parameters tested with the Human Biomarker panel. Cytokine/chemokine levels within human plasma samples from T1D patients and age-matched controls as detected by the Human Biomarker 30-plex V-plex kit (MSD Mesoscale).
(DOCX)
