# Supplementary figures and images for: MIF inhibition interferes with the inflammatory and T cell-stimulatory capacity of NOD macrophages and delays autoimmune diabetes onset
Source: PLoS One. 2017 Nov 2;12(11):e0187455. doi: 10.1371/journal.pone.0187455 (PMC5667746; doi:10.1371/journal.pone.0187455)

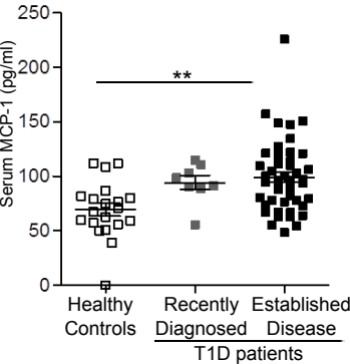

Supplement: S1 Fig — MCP-1 levels were detected in the plasma of established T1D patients (n = 46), recently diagnosed T1D patients (n = 8) and healthy controls (n = 21) as described in the method section. The symbols represent the individual samples tested and show also the mean ± SEM. (PDF) [file pone.0187455.s001.pdf]

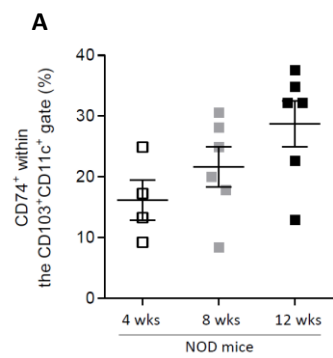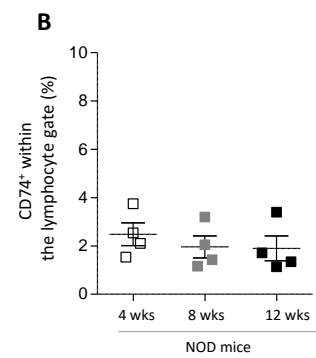

Supplement: S2 Fig — Flow cytometric analysis of homogenized pancreas of NOD mice from different ages whereby the frequency of CD74+ cells within the CD103+CD11c+ dendritic cell-gate (A) or lymphocyte-gate (B) have been analyzed (mean ± SEM; n = 4–6). (PDF) [file pone.0187455.s002.pdf]

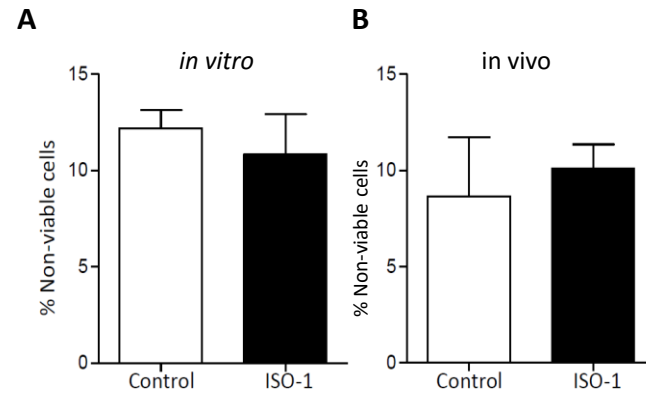

Supplement: S3 Fig — A) Flow cytometric analysis or Live/DEAD staining on macrophages cultured for 24 hours in the presence of ISO-1. Values represent the means ± SEM (n = 4) of the percent non-viable cells. B) Flow cytometric analysis of Live/DEAD staining on macrophages within homogenized pancreas of NOD.SCID animals. The NOD.SCID recipients were adoptively transferred with activated CD4+ T cells obtained from BDC2.5 Tg (1 × 10^5^) and received in vivo treatment with ISO-1 as described in materials and methods. All animals were sacrificed on day 10 post treatment initiation (mean ± SEM; n = 5). (PDF) [file pone.0187455.s003.pdf]

**A**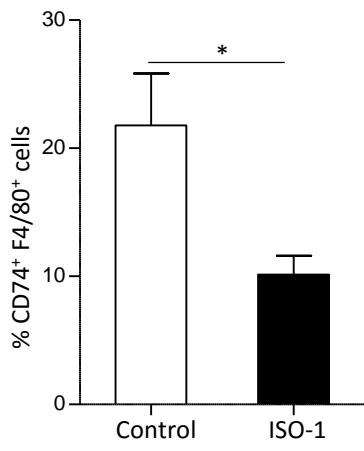**B**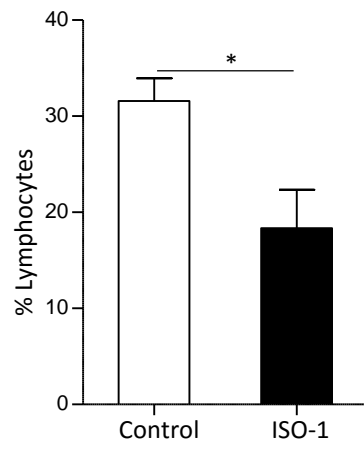

Supplement: S4 Fig — NOD.SCID recipient animals were adoptively transferred with activated CD4+ T cells obtained from BDC2.5 Tg (1 × 10^5^) mice as described in the methods section. The recipient mice received ISO-1 (100 μg; i.p.) (black bars) or vehicle control (white bars) five times a week. On day 10 post treatment initiation, the percentage of CD74+ cells within the F4/80+CD11b+ macrophage population (A) or lymphocytes (B) were quantified in homogenized pancreas samples by flow cytometry (mean ± SEM; n = 5). (PDF) [file pone.0187455.s004.pdf]

C57BL/6

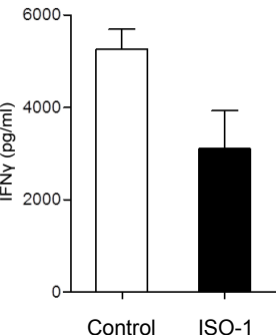

NOD

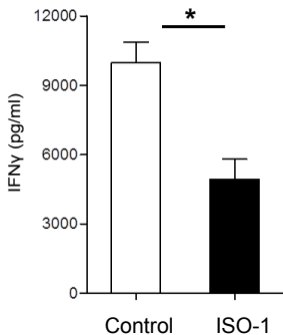

Supplement: S5 Fig — Ctr- or ISO-1-treated macrophages isolated from either C57BL/6 or NOD mice (5 × 10^4^ cells/well) were washed before addition of OVA323-339 peptide or BDC2.5 mimotope (1 μg/mL) and culturing together with negatively isolated CD4+ T cells from OT-II or BDC2.5 Tg mice (1 × 10^5^ cells/well). After 72 hours the supernatants were collected and tested with an MSD IFN-γ V-plex assay. (PDF) [file pone.0187455.s005.pdf]
