# Supplementary material for: MIF inhibition interferes with the inflammatory and T cell-stimulatory capacity of NOD macrophages and delays autoimmune diabetes onset
Source: PLoS One. 2017 Nov 2;12(11):e0187455. doi: 10.1371/journal.pone.0187455 (PMC5667746; doi:10.1371/journal.pone.0187455)
Supplement: S1 Table — The general and disease-associated characteristics of the T1D patients and age-matched controls used to assess circulating MIF levels. (DOCX) [file pone.0187455.s006.docx]

|  | **S1 Table. Patient characteristics.** | | | | |  |
| --- | --- | --- | --- | --- | --- | --- |
|  | | **Healthy Control**  **Group A** | **Recently Diagnosed T1D**  **Group B** | **Established T1D**  **(< 10 years)**  **Group C** | **Established T1D**  **(> 10 years)**  **Group D** | **P value** |
| Cases (n) | | 20 | 37 | 68 | 36 |  |
| Gender (% Male) | | 45 | 60 | 59,42 | 55,26 | n.s. |
| Age (years) | | 27,5 (26 – 33,75) | 28 (23 – 42) | 27,5 (22,25 – 34,75) | 33,5 (27 – 39) | n.s. |
| Disease duration (years) | | - | <1 | 6 (5 – 8) | 17 (13 – 22,75) | *** (C vs D) |
| C-peptide (nmol/l) | | - | 0,32 (0,18 – 0,6) | 0,25 (0,13 – 0.36) | 0,08 (0,01 – 0,18) | n.s. (B vs C); ** (C vs D); *** (B vs D) |
| HbA1c (%) | | - | 11,65 (9,23 – 14,25) | 7,7 (6,7 – 8,6) | 7,65 (7,3 – 8,2) | *** (B vs C); *** (B vs D) |
| ICA (% positive) | | - | 44,44 | 58,33 | 88,89 |  |
| Insulin (% positive) | | - | 22,22 | 19,44 | 66,67 |  |
| GAD65 (% positive) | | - | 51,85 | 61,11 | 55,56 |  |
| I-A2 (% positive) | | - | 33,33 | 47,22 | 55,56 |  |
|  | |  |  |  |  |  |
| The general and disease-associated characteristics of the T1D patients and age-matched controls used to assess circulating MIF levels. #Values represent median (lower quartile – high quartile). **, p < 0.01 vs Control. ***p<0.005 | | | | | | |
