# Supplementary material for: MIF inhibition interferes with the inflammatory and T cell-stimulatory capacity of NOD macrophages and delays autoimmune diabetes onset
Source: PLoS One. 2017 Nov 2;12(11):e0187455. doi: 10.1371/journal.pone.0187455 (PMC5667746; doi:10.1371/journal.pone.0187455)
Supplement: S2 Table — Cytokine/chemokine levels within human plasma samples from T1D patients and age-matched controls as detected by the Human Biomarker 30-plex V-plex kit (MSD Mesoscale). (DOCX) [file pone.0187455.s007.docx]

| **S2 Table. Circulating parameters tested with the Human Biomarker Panel.** | | | |
| --- | --- | --- | --- |
|  | **Controls**  **(n = 21)** | **Recently Diagnosed T1D**  **(n = 8)** | **Established T1D**  **(n = 46)** |
| IFN-γ | 6,1±2,1 | 3,7±0,7 | 4,8±1,4 |
| IL-10 | 0,25±0,1 | 0,3±0,1 | 0,7±0,5 |
| IL12p70 | 0,1±0,03 | 0,3±0,2* | 0,06±0,01 |
| IL-1β | 0,1±0,01 | 0,1±0,02 | 0,1±0,01 |
| IL-6 | 0,2±0,04 | 0,2±0,04 | 0,2±0,03 |
| IL-8 | 4,9±0,3 | 4,3±0,7 | 4,8±0,4 |
| TNFα | 1,3±0,1 | 1,4±0,1 | 1,0±0,04 |
| *Eotaxin* | *89,0**±10,9* | *143,9±29,3* | *168,1±11,2 *** |
| Eotaxin-3 | 11,9±2,0 | 13,9±1,4 | 18,2±2,7 |
| IP-10 | 276,1±31,9 | 348,4±69,8 | 315,3±29,6 |
| *MCP-1* | *69,2**±5,7* | *94,3±6,4* | *99,4±4,9 *** |
| *MCP-4* | *56,8**±7,2* | *87,7±13,7* | *112,2±9,9 *** |
| MDC | 799,1±76,1 | 1014,2±234,0 | 785,0±45,4 |
| MIP-1α | 12,9±1,2 | 14,6±2,1 | 16,2±0,7 |
| MIP-1β | 69,9±9,0 | 110,2±37,2 | 75,0±4,6 |
| TARC | 223,6±30,8 | 338,0±87,7 | 334,1±33,6 |
| GM-CSF | 0,2±0,1 | 0,1±0,1 | 0,06±0,02 |
| IL-12p19 | 112,2±12,4 | 159,4±43,7 | 139,4±10,8 |
| IL-15 | 1,4±0,1 | 2,0±0,5 | 1,5±0,1 |
| IL-16 | 629,6±131,1 | 843,6±392,7 | 513,3±83,3 |
| IL-17A | 1,0±0,2 | 1,1±0,3 | 0,6±0,1 |
| IL-5 | 0,2±0,1 | 0,5±0,3 | 0,2±0,04 |
| *IL-7* | *14,1**±1,9* | *10,2±1,6* | *22,7±1,6*** |
| TNFβ | 0,2±0,1 | 0,3±0,1 | 0,1±0,01 |
| VEGF | 107,2±13,4 | 109,0±22,0 | 156,0±14,8 |
| The values represent cytokine/chemokine levels within human plasma samples from T1D patients and age-matched controls as detected by the Human Biomarker 30-plex V-plex kit (MSD Mesoscale). **, p < 0.01 vs Control | | | |
